# Serum level of adiponectin is a surrogate independent biomarker of radiographic disease progression in early rheumatoid arthritis: results from the ESPOIR cohort

**DOI:** 10.1186/ar4404

**Published:** 2013-12-09

**Authors:** Magali Meyer, Jérémie Sellam, Soraya Fellahi, Salma Kotti, Jean-Philippe Bastard, Olivier Meyer, Frédéric Lioté, Tabassome Simon, Jacqueline Capeau, Francis Berenbaum

**Affiliations:** 1 Department of Rheumatology, AP-HP, Saint-Antoine Hospital, Inflammation-Immunopathology-Biotherapy i2B Department, 184, rue du Faubourg Saint-Antoine, 75012 Paris, France; 2 UPMC Univ Paris 06, F-75005 Paris, France; 3 INSERM, UMR_S 938, Faculté de Médecine Saint Antoine, F-75012 Paris, France; 4 Department of Biochemistry, AP-HP, Hopital Tenon, F-75020 Paris, France; 5 Unité de Recherche Clinique de l’Est Parisien, AP-HP, Hôpital Saint-Antoine, Paris, France; 6 Department of Rheumatology, Paris Diderot Paris VII University, AP-HP, Bichat Hospital, Paris, France; 7 Department of Rheumatology, Paris Diderot Paris VII University, AP-HP, Lariboisière Hospital, Paris, France

## Abstract

**Introduction:**

Adipokines such as adiponectin, leptin, and visfatin/nicotinamide phosphoribosyltransferase (NAMPT) have recently emerged as pro-inflammatory mediators involved in the pathophysiology of rheumatoid arthritis (RA). We aimed to determine whether serum adipokine levels independently predicted early radiographic disease progression in early RA.

**Methods:**

In total, 791 patients were included from the prospective Etude et Suivi des POlyarthrites Indifférenciées Récentes (ESPOIR) cohort who met the American College of Rheumatology-European League Against Rheumatism criteria for RA (*n* = 632) or had undifferentiated arthritis (UA) (*n* = 159). Enzyme-linked immunosorbent assay (ELISA) was used to assess baseline serum levels of adiponectin, leptin, and visfatin/NAMPT. In the RA group, we tested the association of serum adipokine levels and (a) baseline radiographic damage and (b) radiographic disease progression, defined as a change >0 or ≥5 in total Sharp-van der Heijde Score (∆SHS) between inclusion and 1 year (∆SHS ≥1 or rapid radiographic progression: ∆SHS ≥5), adjusting for confounders (age, sex, body-mass index, insulin resistance, C-reactive protein level, Disease Activity Score in 28 joints, Health Assessment Questionnaire score, autoantibody status, steroid use, and radiographic evidence of RA damage at inclusion).

**Results:**

Adiponectin level was independently associated with baseline total SHS (adjusted β = 0.12; *P* = 0.006). It was also associated with ∆SHS ≥1 (adjusted odds ratio (aOR) = 1.84 (1.25 to 2.72)) involving erosive as well as narrowing disease progression (aOR = 1.73 (1.17 to 2.55) and 1.93 (1.04 to 3.57), respectively). Serum adiponectin level predicted ∆SHS ≥5 (aOR = 2.0 (1.14 to 3.52)). Serum leptin level was independently associated only with ∆SHS >0 (aOR = 1.59 (1.05 to 2.42)). Conversely, serum visfatin/NAMPT level and radiographic disease progression were unrelated. Considering the receiver-operated characteristic curves, the best adiponectin cut-offs were 4.14 μg/ml for ∆SHS ≥1 and 6.04 μg/ml for ∆SHS ≥5, with a good specificity (58% and 75% for ∆SHS ≥1 and ∆SHS ≥5, respectively) and high negative predictive values (75% and 92% for ∆SHS ≥1 or ∆SHS ≥5, respectively).

**Conclusion:**

Serum adiponectin level is a simple useful biomarker associated with early radiographic disease progression in early RA, independent of RA-confounding factors and metabolic status.

## Introduction

In rheumatoid arthritis (RA), early diagnosis and early therapeutic intervention improve clinical outcomes and reduce the accrual of joint damage and major irreversible long-term disability. Patients with early RA should be actively treated as soon as the disease is diagnosed [1]. Finding serum biomarkers that can be used to identify patients at high risk of structural disease progression early in the disease is important because joint lesions occur mainly during this period and may be reduced by early treatment [2]. Levels of inflammatory biomarkers (that is, C-reactive protein (CRP) and erythrocyte sedimentation rate (ESR)) and presence of autoantibodies (that is, rheumatoid factor (RF) and anti-cyclic citrullinated peptide [anti-CCP] antibodies) are associated with subsequent RA structural severity [3-5]. However, the predictive value of these markers for early radiographic disease progression remains weak, especially in early disease, which supports the need for more sensitive and specific biomarkers [6].

Adipokines are adipose tissue-specific soluble proteins also produced by synovium and cartilage, as well as mononuclear blood cells; they are quantifiable in biologic fluids [7]. The most studied are adiponectin, leptin, and visfatin (also known as nicotinamide phosphoribosyltransferase (NAMPT)). These soluble mediators have pleiotropic effects and participate in several metabolic, immune, and inflammatory processes [7-9]. Adiponectin is the most abundant adipokine detected in the serum and has unique properties by acting as an antidiabetic and antiatherogenic mediator [10,11]. However, despite the cardioprotective properties of adiponectin and considering the complexity of its effects, a high serum level of adiponectin was found to be associated with mortality in kidney diseases and type 1 diabetes; this increased level is paradoxically considered as a beneficial physiological response [10-14]. In type 1 diabetes, increased leptin levels are positively associated with β-cell function [15]. In nonrheumatic conditions, serum ratio of adiponectin to leptin may be a noninvasive marker of the severity of nonalcoholic fatty liver disease [16].

The important role of adipokines in inflammation provides novel links between adipose tissue, adipokines, and inflammation-related disorders, including RA [11]. Therefore, adipokines could represent potential disease biomarkers and therapeutic targets in clinical practice. Recent findings have demonstrated that these mediators have potent actions on tissue and cells involved in RA, including cartilage, synovium, bone, and various immune cells [17]. Adipokines as serum biomarkers reflecting RA-related radiographic damage were recently reported in two cross-sectional studies of 167 and 197 subjects [18,19]. Moreover, serum adiponectin level as a predictor of radiographic disease progression has been suggested in two longitudinal studies of 152 patients with established RA and 253 with early RA [20,21]. However, in all these studies, several important confounding factors, such as metabolic disorders, especially insulin resistance, were not taken into account.

Further to assess the role of adipokines as serum biomarkers for early radiographic disease progression in RA, we determined in a large cohort of patients with early arthritis whether serum adipokine levels (a) were increased in early RA as compared with undifferentiated arthritis (UA), and (b) could predict radiographic structural progression in patients with early RA, and especially rapid radiographic progression.

## Methods

### Patients

The population and methods of the ESPOIR cohort (Etude et Suivi des POlyarthrites Indifférenciées Récentes) were described in detail elsewhere [22]. Diagnosis of RA was defined as fulfilling the American College of Rheumatology-European League Against Rheumatism (ACR-EULAR) 2010 criteria for RA at inclusion [1]. Otherwise and with no other defined diagnosis at inclusion, patients were considered to have UA. Patients with other defined diagnoses were not assessed for serum adipokine level. The Montpellier (France) Ethics Committee approved the study in July 2002, and all patients provided informed consent.

### Serum assays

At inclusion, blood samples were taken for investigation of glycemia, C-reactive protein (CRP) level, and erythrocyte sedimentation rate (ESR). Serum samples were collected at enrolment and immediately stored at -80°C in a single biologic resource centre. A central laboratory was used for determining anti-citrullinated cyclic peptide (anti-CCP) antibodies (anti-CCP2; Dia-Sorin, Saluggia (Vercelli), Italy; positive >50 U/ml) and rheumatoid factor (RF) (Menarini France, Rungis Cedex, France; positive >9 IU/ml) with enzyme-linked immunosorbent assay (ELISA), as previously described [23]. ELISA kits were used for assay of serum levels of total adiponectin (Bühlman, Basel, Switzerland), leptin (Quantikine; R&D Systems, Oxford, UK), and visfatin/NAMPT (Bühlman, Basel, Switzerland). Insulinemia, used for calculation of the Homeostatic Model Assessment of Insulin Resistance (HOMA-IR) index, was assayed with chemiluminescence (ARCHITECT Insulin, Abbott Park, IL, USA).

### Radiologic data

Radiographs of the hands and wrists (anteroposterior view) and the feet (anteroposterior and oblique views) were available at inclusion and at 1 year. The interpretation was standardized, as described previously [22,24]. Radiographs were read by the patient’s office-based rheumatologist to determine the presence or absence of abnormalities related to RA. Then, for quantitative scoring, radiographs were sent to the coordinating center for calculation of the total, joint erosion, and narrowing Sharp/van der Heijde score (SHS) at inclusion and 1 year by two experienced readers with blinding to patient data. Missing data were excluded from the analysis.

### Statistical analysis

Continuous variables are described as mean ± standard deviation (SD), and categoric variables as frequencies and percentages. The distributions of all variables were examined. Serum levels of adiponectin, leptin, visfatin/NAMPT, and CRP, Health Assessment Questionnaire (HAQ) score, and SHS values were not normally distributed and thus were log-transformed to remove positive skewness.

Baseline demographic, clinical, and radiographic characteristics of RA and UA patients were compared with χ^2^ or Fisher Exact tests for discrete variables, and unpaired *t* test or Kruskal-Wallis test for continuous variables.

The association of adipokine levels and baseline radiographic damage was tested by univariate and multivariate linear regression analyses for patients with SHS >0. Patients with SHS = 0 were removed from these analyses after logarithmic transformation.

Univariate and multivariate logistic regression analyses were used to model radiographic progression as a dichotomous variable, with the primary outcome defined by any change in total SHS (∆SHS ≥1), as previously examined in the ESPOIR cohort [25,26]. Similarly, erosive and narrowing disease progression was defined as a ∆SHS ≥1 in erosive and narrowing subscores, respectively. Additional analyses involved a stricter outcome of any increase in total SHS ≥5 (∆SHS ≥5), which delineates the subgroup of patients with rapid radiographic disease progression, also previously examined in the ESPOIR cohort [27].

Relevant known confounders were included in multivariate analyses models. We consider four models as follows: model 1, or “base” model, adjusted on age and sex; Model 2, or “metabolic” model, adjusted on Model 1 variables and body mass index (BMI) and HOMA-IR index; model 3, or “metabolic and RA” model, adjusted on model 2 variables and CRP level, DAS28, HAQ score, RF status, anti-CCP antibody status, and, for the association with radiographic progression, presence of typical damage related to RA at inclusion; and model 4, or “metabolic, RA, and steroid” model, adjusted on model 3 variables and steroid prescription at inclusion.

Sensitivity versus the false-positive frequency (1-specificity) for predicting radiographic disease progression (∆SHS ≥1 or ≥5) with adipokines levels and with other markers of radiographic disease progression (that is, DAS28 value, number of swollen joints, CRP level, RF level, and anti-CCP antibodies level) was analyzed with a receiver-operated characteristic (ROC) curve. The predictive accuracy of each item was assessed by using the area under the curve (AUC). To determine the optimal cut-off of adiponectin level, the Youden index was calculated by using the following formula: sensitivity + specificity – 1, and the maximum value of the Youden index corresponded to the optimal cut-off point [28].

All statistical tests were two-sided with a statistical significance defined as *P* < 0.05. SAS v9.3 (SAS Inst., Cary, NC, USA) was used for analysis.

## Results

### Characteristics of the study population and comparison of RA and UA patients

Among 813 patients enrolled in the whole cohort, 791 had data for serum adipokine levels and were analyzed in this study; 632 had RA fulfilling the ACR-EULAR 2010 criteria, and 159 had UA. Comparisons of clinical, biologic, and radiologic characteristics of RA and UA patients are shown in Table 1. At inclusion, RA patients were more often positive for anti-CCP antibodies and RF and showed radiographic changes than did UA patients. In addition, DAS28, HAQ score, and CRP level were significantly higher for RA than for UA patients. Baseline total SHS was higher for RA than for UA patients, the difference involving predominantly the erosive subscore. At 1-year follow-up, similar results were observed, with a wider variation of total and erosive SHS in the RA group.

**Table 1:** Characteristics of patients with early rheumatoid arthritis (RA) and undifferentiated arthritis (UA) from the ESPOIR cohort

	**UA (*n*** **= 159)**	**Early RA (*n*** **= 632)**	***P *** **value**
**Age**	47.2 ± 13.8	48.5 ± 12.2	0.46
**Women ( *n *,%)**	117 (74%)	492 (78%)	0.25
**First symptom (months)**	6.6 ± 7.7	6.9 ± 8.5	0.72
**DAS28**	4.0 ± 1.0	5.4 ± 1.2	**<0.0001**
**CRP level (mg/L)**	17.2 ± 29.3	21.1 ± 33.1	**0.0028**
**ESR**	25.3 ± 22.4	30.6 ± 24.9	**0.0014**
**Positive anti-CCP antibodies ( *n *,%)**	2 (1.26%)	313 (49.5%)	**<0.0001**
**Positive RF ( *n *,%)**	5 (3.1%)	365 (57.75%)	**<0.0001**
**Swollen-joint count**	3.5 ± 2.4	8.2 ± 5.2	**<0.0001**
**Tender-joint count**	3.2 ± 2.6	9.9 ± 7.2	**<0.0001**
**HAQ score**	0.69 ± 0.58	1.05 ± 0.69	**<0.0001**
**Diabetes mellitus ( *n *,%)**	7 (4.4%)	24 (3.8%)	0.73
**HOMA-IR index**	2.5 ± 2.6	2.8 ± 4.2	0.37
**BMI**	24.7 ± 4.6	25.2 ± 4.6	0.22
**Steroid prescription at inclusion ( *n *,%)**	22 (13.8%)	84 (13.3%)	0.86
**Radiographic damage at inclusion ( *n *,%)**	0 (0)	108 (17.1%)	**<0.0001**
**Total SHS at inclusion**	4.6 ± 6.8	6.14 ± 7.92	**0.005**
**Erosive SHS**	1.6 ± 3.13	3.09 ± 4.98	**<0.0001**
**Narrowing SHS**	2.97 ± 4.82	3.04 ± 4.36	0.77
**Total SHS at 1 year**	5.7 ± 9.5	7.7 ± 10.7	**0.006**
**Erosive SHS**	2.35 ± 5.05	4.48 ± 7.56	**<0.0001**
**Narrowing SHS**	3.36 ± 5.54	3.26 ± 4.78	0.9552
**Total ∆SHS between inclusion and 1 year**	1.0 ± 3.8	1.6 ± 4.4	**0.02**
**Erosive ∆SHS between inclusion and 1 year**	0.73 ± 2.7	1.37 ± 3.73	**0.0025**
**Narrowing ∆SHS between inclusion and 1 year**	0.24 ± 1.22	0.26 ± 1.15	0.7294
**Adiponectin level (μg/ml)**	4.9 ± 3.4	5.0 ± 3.7	0.63
**Leptin level (ng/ml)**	14.6 ± 14.4	14.4 ± 13.7	0.74
**Visfatin/NAMPT level (ng/ml)**	4.1 ± 3.6	3.57 ± 3.1	0.47

Data are expressed as mean ± SD unless indicated. Bold *P* values are significant.

DAS28, Disease Activity Score in 28 joints; CRP, C-reactive protein level; ESR, erythrocyte sedimentation rate; anti-CCP, anti-cyclic citrullinated protein peptide antibodies; RF, rheumatoid factor; HAQ, Health Assessment Questionnaire; VAS, visual analog scale; BMI, body mass index; HOMA-IR, Homeostatic Model Assessment of Insulin Resistance, SHS, total Sharp/van der Heijde score; ∆SHS, variation in SHS between inclusion and 1 year.

Despite these differences, serum adipokine levels did not significantly differ between RA and UA patients (Table 1). However, adiponectin levels were higher for RA patients with positivity for anti-CCP antibodies or RF than for those without (5.22 ± 3.96 versus 4.58 ± 3.29 μg/ml; *P* = 0.04). Leptin level was higher but not significantly for patients without than for those with auto-antibodies (13.75 ± 12.70 versus 15.47 ± 15.35 ng/ml; *P* = 0.07). Conversely, visfatin/NAMPT level did not vary by auto-antibody status (*P* = 0.59).

### Serum adipokine levels and structural damage for patients with RA at baseline

Baseline serum adiponectin level was associated with baseline total SHS even after adjustment for metabolic and RA confounders, as well as steroid prescription (model 4: β = 0.12; *P* = 0.006) (Table 2). This association involved mainly the joint-narrowing subscore (model 4: β = 0.14; *P* = 0.01). Conversely, leptin and visfatin/NAMPT levels were not associated with total SHS on univariate or multivariate analysis (Table 2).

**Table 2:** **Unadjusted and adjusted association of serum adipokine levels and total joint erosive and narrowing Sharp/van der Heijde score (SHS) at inclusion for RA patients with SHS ≥1 (*n*** **= 446)**

		**Univariate**		**Model 1 “Base”**		**Model 2 “metabolic”**		**Model 3 “metabolic + RA”**		**Model 4 “metabolic + RA + steroid”**	
		**β**	***P***	**β**	***P***	**β**	***P***	**β**	***P***	**β**	**p**
**Adiponectin**	**Total SHS**	0.058	0.1	0.097	**0.007**	0.1	**0.006**	0.11	**0.007**	0.12	**0.006**
	**Erosive SHS**	-0.004	0.93	0.035	0.40	0.03	0.52	0.06	0.22	0.06	0.22
	**Narrowing SHS**	0.097	**0.03**	0.1	**0.02**	0.13	**0.006**	0.14	**0.009**	0.14	**0.01**
**Leptin**	**Total SHS**	0.015	0.75	0.015	0.73	-0.01	0.79	-0.01	0.76	-0.01	0.74
	**Erosive SHS**	-0.15	**0.005**	-0.13	**0.01**	-0.09	**0.02**	-0.06	0.14	-0.07	0.13
	**Narrowing SHS**	0.13	**0.02**	0.11	**0.047**	0.04	0.40	0.02	0.64	0.03	0.63
**Visfatin/NAMPT**	**Total SHS**	0.006	0.89	0.008	0.87	-0.01	0.82	-0.04	0.46	-0.04	0.46
	**Erosive SHS**	-0.08	0.11	-0.1	0.06	-0.12	0.03	-0.14	**0.02**	-0.14	**0.02**
	**Narrowing SHS**	-0.004	0.95	0.02	0.70	-0.009	0.89	-0.02	0.71	-0.02	0.71

Multivariate models: Model 1 (“base”): sex + age. Model 2 (“metabolic”): Model 1 + BMI + HOMA-IR index. Model 3 (“metabolic + RA”): Model 2 + CRP level + DAS28-ESR value + HAQ score + RF status + anti-CCP antibody status. Model 4 (“metabolic + RA + steroid”): Model 3 + steroid prescription at inclusion. The following variables were log-transformed: CRP level, HAQ score, serum adipokine levels. Results are presented as β regression coefficient and *P* value. Bold *P* values are significant.

### Association of adipokine levels and radiographic disease progression

**Figure 1:**
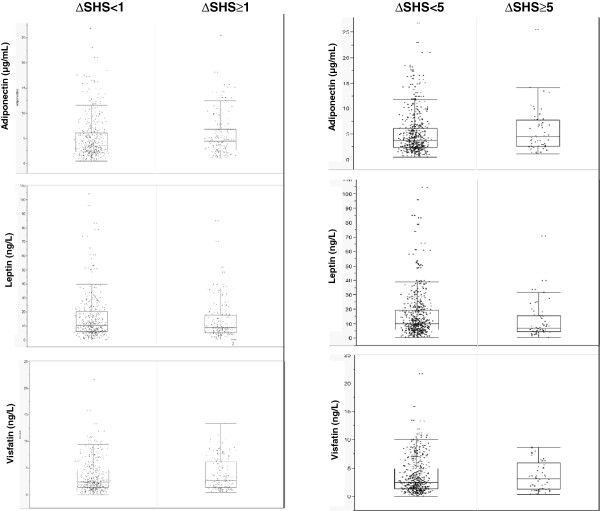
Dot blots and outlier boxplots of baseline serum adipokines levels according to the radiographic disease progression (∆SHS ≥1 versus ∆SHS <1 and ∆SHS ≥5 versus ∆SHS <5) in early RA patients.

Serum level of each adipokine are graphically reported in the Figure 1 according to the presence (n=160) or not of radiographic progression at 1 year (that is, ∆SHS ≥1 or ∆SHS <1).

**Figure 2:**
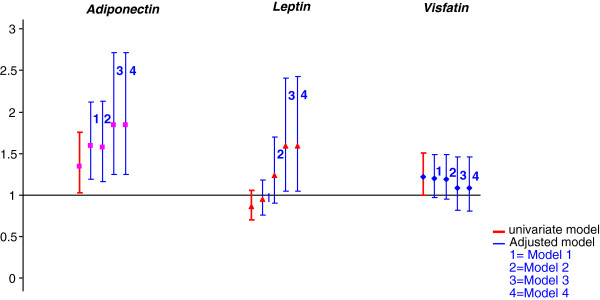
**Association of baseline serum adipokine levels and radiographic evidence of disease progression defined as an increase of ≥1 in total Sharp/van der Heijde score (SHS) between inclusion and 1 year.** Multivariate models: model 1 (“base”): sex + age. Model 2 (“metabolic”): model 1 + BMI + HOMA-IR index. Model 3 (“metabolic + RA”): model 2 + CRP level + DAS28-ESR value + HAQ score + RF status + anti-CCP antibody status + presence of radiographic changes at inclusion. Model 4 (“metabolic + RA + steroid”): model 3 + steroid prescription at inclusion. DAS28, Disease Activity Score in 28 joints; CRP, C-reactive protein level; ESR: erythrocyte sedimentation rate; RF, rheumatoid factor; HAQ, Health Assessment Questionnaire; BMI, body mass index; HOMA-IR, Homeostatic Model Assessment of Insulin Resistance.

Baseline serum adiponectin level was associated with radiographic disease progression (∆SHS ≥1) in both univariate and multivariate models (Table 3, Figure 2). In Model 3 (“metabolic and RA”), baseline serum adiponectin level and radiographic progression at 1 year were related (adjusted odds ratio (OR), 95% confidence interval = 1.84 (1.24 to2.71), *P* = 0.002). In other words, with each 1-unit increase in log(adiponectin) level, the risk of radiographic progression at 1 year was increased 84%. This association persisted after adjustment for steroid prescription at inclusion (Model 4 in Table 3 and Figure 2).

**Table 3:** Association of baseline serum adipokine levels and radiographic disease progression defined as an increase of ≥1 in total SHS between inclusion and 1 year

		**Univariate model**		**Model 1 “Base”**		**Model 2 “Metabolic”**		**Model 3 “Metabolic + RA”**		**Model 4 “Metabolic + RA + steroid”**	
		**OR (95% CI)**	***P***	**OR (95% CI)**	***P***	**OR (95% CI)**	***P***	**OR (95% CI)**	***P***	**OR (95% CI)**	***P***
**Adiponectin**	**Total ∆SHS ≥1**	1.34 (1.03-1.76)	0.03	1.59 (1.19-2.12)	0.002	1.57 (1.16-2.13)	0.004	1.84 (1.24-2.71)	0.002	1.84 (1.25-2.72)	**0.002**
	**Erosive ∆SHS ≥1**	1.34 (1.02-1.75)	0.03	1.56 (1.17-2.08)	0.003	1.54 (1.13-2.08)	0.006	1.73 (1.17-2.55)	0.006	1.73 (1.17-2.55)	**0.006**
	**Narrowing ∆SHS ≥1**	1.65 (1.05-2.61)	0.03	1.79 (1.10-2.90)	0.02	1.95 (1.16-3.27)	0.01	1.91 (1.04-3.52)	0.04	1.93 (1.04-3.57)	**0.04**
**Leptin**	**Total ∆SHS ≥1**	0.86 (0.71-1.05)	0.14	0.95 (0.76-1.18)	0.63	1.24 (0.9-1.70)	0.2	1.59 (1.05-2.41)	0.03	1.59 (1.05-2.42)	**0.03**
	**Erosive ∆SHS ≥1**	0.88 (0.72-1.07)	0.19	0.96 (0.77-1.20)	0.69	1.29 (0.93-1.78)	0.12	1.68 (1.11-2.56)	0.01	1.69 (1.11-2.57)	**0.01**
	**Narrowing ∆SHS ≥1**	0.93 (0.66-1.3)	0.66	0.93 (0.64-1.35)	0.70	1.09 (0.63-1.87)	0.76	1.1 (0.58-2.09)	0.76	1.10 (0.58-2.08)	0.77
**Visfatin/NAMPT**	**Total ∆SHS ≥1**	1.22 (0.99-1.51)	0.06	1.20 (0.97-1.49)	0.09	1.2 (0.95-1.49)	0.12	1.09 (0.81-1.46)	0.57	1.09 (0.81-1.46)	0.58
	**Erosive ∆SHS ≥1**	1.25 (1.01-1.54)	0.04	1.23 (0.99-1.52)	0.06	1.22- (0.98-1.53)	0.08	1.14 (0.85-1.53)	0.37	1.14 (0.85-1.54)	0.37
	**Narrowing ∆SHS ≥1**	1.17 (0.82-1.67)	0.39	1.16 (0.81-1.67)	0.41	1.05 (0.73-1.53)	0.79	1.12 (0.72-1.75)	0.62	1.13 (0.72-1.77)	0.6

OR, odds ratio; 95% CI, 95% confidence interval. Total patient number, 530.

Data are unadjusted and adjusted odds ratios with p values. Bold *P* values are significant.

Radiographic progression is defined as variation in total SHS between inclusion and 1 year (total ∆SHS) ≥1.

Erosive radiographic progression is defined as variation in erosive SHS between inclusion and 1 year (erosive ∆SHS ≥1).

Narrowing radiographic progression is defined as variation in narrowing SHS between inclusion and 1 year (narrowing ∆SHS ≥1).

Multivariate models:

Model 1 (“base”): sex + age (*n* = 530).

Model 2 (“metabolic”): Model 1 + BMI + HOMA-IR index (*n* = 497).

Model 3 (“metabolic + RA”): Model 2 + CRP level + DAS28-ESR value + HAQ score + RF status + anti-CCP antibody status + presence of radiographic changes at inclusion (*n* = 344).

Model 4 (“metabolic + RA + steroid”): Model 3 + steroid prescription at inclusion (*n* = 344).

The following variables were log-transformed: CRP level, HAQ score, serum adipokine levels.

Similarly, baseline serum adiponectin level was associated with erosive and narrowing disease progression (defined as ∆SHS erosive subscore ≥1 and ∆SHS narrowing subscore ≥1, respectively): adjusted (model 3) OR = 1.73 (1.17 to 2.55) (*P* = 0.006) and 1.91 (1.04 to 3.52) (*P* = 0.038), respectively (Table 3 and Additional file 1: Figure S1). This association persisted even after adjustment for steroid prescription at inclusion. No interaction was seen between adiponectin level and the two other adipokines levels, and the predictive value of adiponectin did not change when serum leptin and visfatin/Nampt were included in the model 4 (data not shown).

We strengthened our results with a more-stringent definition of radiographic disease progression (∆SHS ≥5; n=54). Serum levels of each adipokine according to the presence or absence of rapid radiographic progression are graphically reported in Figure 1. We still found an association of baseline serum adiponectin level and rapid radiographic progression even after adjustment for all considered confounders (model 4) (OR, 2.0 (1.14 to 3.52), *P* = 0.02) (Table 4, Figure 2).

**Table 4:** Association of baseline serum adipokine levels and rapid radiographic disease progression defined as an increase of ≥ 5 units in total SHS between inclusion and 1 year (total ∆SHS ≥5)

		**Univariate model**		**Model 1 “Base”**		**Model 2 “metabolic”**		**Model 3 “metabolic + RA”**		**Model 4 “metabolic + RA + steroid”**	
		**OR (95% CI)**	***P***	**OR (95% CI)**	***P***	**OR (95% CI)**	***P***	**OR (95% CI)**	***P***	**OR (95% CI)**	***P***
**Adiponectin**	**∆SHS ≥ 5**	1.47 (0.98-2.20)	0.06	1.7 (1.11-2.61)	**0.02**	1.85 (1.16-2.94)	**0.01**	2.0 (1.14-3.52)	**0.02**	2.0 (1.14-3.52)	**0.02**
**Leptin**	**∆SHS ≥ 5**	0.73 (0.54-0.97)	**0.03**	0.75 (0.55-1.04)	0.09	0.98 (0.61-1.56)	0.92	0.97 (0.55-1.68)	0.90	0.97 (0.56-1.68)	0.90
**Visfatin/NAMPT**	**∆SHS ≥ 5**	1.14 (0.83 1.56)	0.41	1.12 (0.82-1.54)	0.48	1.11 (0.79-1.55)	0.56	0.96 (0.64-1.44)	0.85	0.96 (0.64-1.44)	0.85

OR, odds ratio; 95% CI, 95% confidence interval.

Multivariate models:

Model 1 (“base”): sex + age.

Model 2 (“metabolic”): model 1 + BMI + HOMA-IR index.

Model 3 (“metabolic + RA”): model 2 + CRP level + DAS28-ESR value + HAQ score + RF status + anti-CCP antibody status + presence of radiographic changes at inclusion.

Model 4 (“metabolic + RA + steroid”): model 3 + steroid prescription at inclusion.

The following variables were log-transformed: CRP level, HAQ score, serum adipokine levels. Bold *P* values are significant.

**Figure 3:**
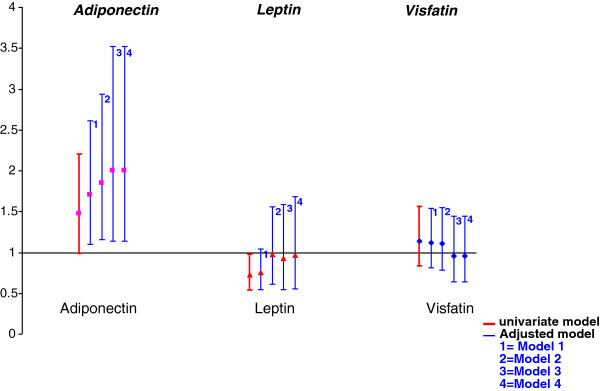
**Association of baseline serum adipokine levels and radiographic disease progression defined as an increase of ≥5 in total SHS between inclusion and 1 year.** Multivariate models: Model 1 (“base”): sex + age. Model 2 (“metabolic”): model 1 + BMI + HOMA-IR index. Model 3 (“metabolic + RA”): model 2 + CRP level + DAS28-ESR value + HAQ score + RF status + anti-CCP antibody status + presence of radiographic changes related to RA at inclusion. Model 4 (“metabolic + RA + steroid”): model 3 + steroid prescription at inclusion. DAS28, Disease Activity Score in 28 joints; CRP, C-reactive protein level; ESR, erythrocyte sedimentation rate; RF, rheumatoid factor; HAQ, Health Assessment Questionnaire; BMI, body mass index; HOMA-IR, Homeostatic Model Assessment of Insulin Resistance.

For leptin level, with radiographic disease progression considered as ∆SHS ≥1 and after controlling on RA-related and metabolic parameters, with each 1-unit increase in log(leptin) level, the risk of radiographic progression at 1 year was increased 59% (model 3 or 4, OR = 1.59 (1.05 to 2.41); *P* = 0.03) (Table 3, Figure 2). This association was exclusively to the result of erosive disease progression (model 3 OR, 1.68 [1.1 to 2.56]; *P* = 0.01) (Table 3 and Additional file 1: Figure S1). These results did not change after controlling for steroid prescription (model 4). The association of leptin level and radiographic progression disappeared after adjustment when considering disease progression as ∆SHS ≥5 (Table 4, Figure 3).

**Figure 4:**
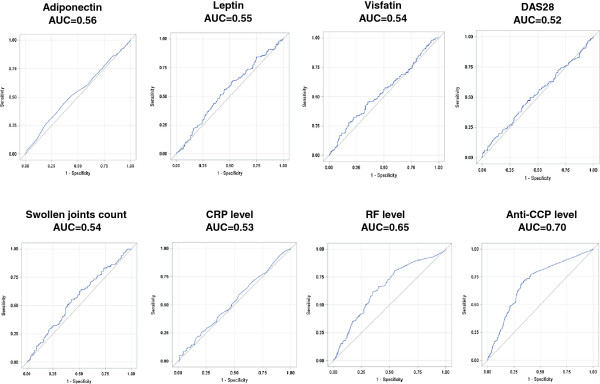
**Receiver operating characteristic (ROC) curves for predicting radiographic progression (ΔSHS ≥1).** The corresponding value of area under the curve (AUC) is reported for each item.

**Figure 5:**
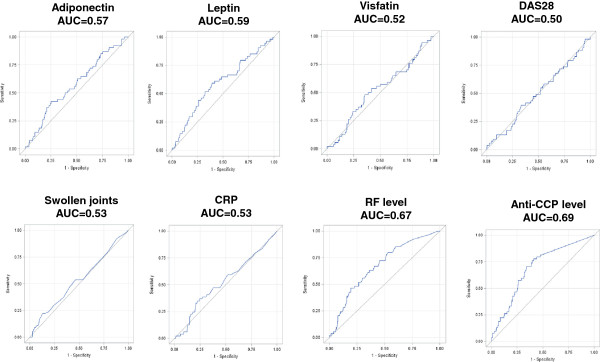
**Receiver operating characteristic (ROC) curves for predicting radiographic progression (ΔSHS ≥5).** The corresponding value of area under the curve (AUC) is reported for each item.

To estimate the accuracy of serum leptin and serum adiponectin to independent prediction of radiographic disease progression at 1 year (∆SHS ≥1 or ∆SHS ≥5), we performed ROC curves analyses of these two adipokines and also of other known markers associated with radiographic disease progression or rapid radiographic disease progression (/that is, DAS28 value, number of swollen joints, CRP level, RF level, and ACPA level) (Figure 4 for ∆SHS ≥1 and Figure 5 for ∆SHS ≥5). Interestingly, the AUC of each ROC curve of serum adiponectin and serum leptin was in the same range as the AUC of baseline DAS28, number of swollen joints, or CRP level (*P* values nonsignificant). As expected and because of the accuracy of autoantibodies, RF level and anti-CCP antibodies level displayed higher AUC (*P* values <0.05).

Considering the higher values of sensitivity combined with the lower values (1-specificity), the best cut-off for serum adiponectin level was 4.14 μg/ml (sensitivity, 0.55, and specificity, 0.58) and 6.04 μg/ml (sensitivity, 0.43, and specificity, 0.75) to predict ∆SHS ≥1 or ≥5, respectively. We also calculated the negative predictive value (75% and 92% for ∆SHS ≥1 or ∆SHS ≥5, respectively) and the positive predictive value (36% and 16% for ∆SHS ≥1 or ∆SHS ≥5, respectively), suggesting that a nonelevation of serum adiponectin beyond the threshold may imply that the risk of radiographic disease progression is very low.

We used linear regression analyses to investigate the association of baseline serum adipokine levels and total SHS at 1 year to ensure the association of serum adiponectin or leptin levels and radiographic disease progression. We focused on patients with severe disease, with prevalent radiographic lesions, because the log-transformed SHS automatically excluded all patients with SHS of 0. Again, serum adiponectin level predicted radiographic disease damage at 1 year (model 4, β = 0.13, *P* = 0.0026; Additional file 2: Table S1). Conversely, leptin level was not associated with total SHS at 1 year.

Baseline visfatin/NAMPT levels did not predict radiographic disease progression in any models, whatever the definition of radiographic disease progression used.

## Discussion

We used data for the French ESPOIR cohort, one of the largest worldwide cohorts of early RA patients followed up longitudinally. We demonstrated that, in patients fulfilling the ACR-EULAR 2010 criteria for early RA at inclusion in the cohort, serum level of adiponectin is associated with subsequent early radiographic disease progression, independent of several RA and metabolic confounders and especially with rapid radiographic disease progression (∆SHS ≥5). Serum adiponectin level is associated with both erosive and narrowing subscores of radiographic disease progression.

Klein-Wieringa *et al*. [1,20] investigated adipokine levels in 253 patients with early RA by using the 1987 ACR criteria, but we chose the new ACR-EULAR 2010 criteria, which do not involve radiographic results and thus are more accurate for early RA diagnosis and more adapted to identify new biomarkers of RA severity early during the disease course.

Several cross-sectional studies of established RA have shown a positive association of serum levels of adiponectin and visfatin/NAMPT and an inverse association of serum leptin level and radiographic joint damage [18,19]. However, we found that baseline serum adiponectin level was associated with quantitative joint damage, as assessed by SHS, mainly the joint-narrowing SHS subscore. Conversely, serum leptin was not associated with radiographic RA damage after multiple adjustments. Several reasons may explain such discrepancies. First, characteristics of the population differed because we investigated a large cohort of patients with early, not-established, untreated RA, whereas Rho *et al.*[19] investigated 167 patients with established RA of 3-year duration. Second, we analyzed RA patients with SHS ≥1, characterized by a more severe structural disease at inclusion, because of the logarithmic transformation of SHS necessary to meet the assumption of linear regression analysis. We found no association of leptin level and radiographic progression by total SHS; this finding is in agreement with that of Rho *et al.*[19], who showed that the association of serum leptin level and radiographic damage did not persist after adjustment for BMI, considered an important confounder.

Several of our statistical analyses show that adiponectin and leptin can predict radiographic progression and thus disease severity. Although adiponectin could have protective roles in some tissues [8], preclinical studies have demonstrated its deleterious role in cartilage homeostasis. Adiponectin induces the release of proinflammatory cytokines (that is, IL-6, monocyte chemotactic protein-1 (MCP-1)), pro-degradative enzymes (that is, matrix metalloproteinases (MMPs)) and nitric oxide in chondrocytes [29-33]. These experimental data may explain, at least in part, a link between serum adiponectin level and joint-space narrowing found in our study. Moreover, synovial fibroblasts express adiponectin receptors and are reactive to adiponectin stimulation, triggering a proinflammatory and prodegradative state characterized by prostaglandin E_2_, IL-6, IL-8, MCP-1, and MMPs releases [29,33,34].

Furthermore, the paradoxic adiponectin effects between the cardiovascular system and joints may be related to its different isoforms. However, all adiponectin isoforms were shown to exert a proinflammatory and joint-destructive role on synovial cells [35]. Moreover, we previously compared levels of total and of the main isoform of adiponectin, “high-molecular-weight” (HMW) adiponectin in a large number of individuals with a wide range of BMI (*n* = 408). We found that total adiponectin and HMW-adiponectin were highly correlated (*r* = 0.927; *P* < 0.001, JP Bastard, personal communication). Therefore, total serum adiponectin level could be considered a good reflection of the HMW-adiponectin level. However, the direct assessment in the serum of the several adiponectin isoforms remains to be investigated in RA to compare its accuracy with serum total adiponectin level to predict structural progression.

Giles *et al.*[21] performed a cross-sectional study assessing the association of serum adipokine levels and radiographic damage and showed an association of high adiponectin level and erosions, as well as joint narrowing. Our study is the first to investigate the longitudinal association of serum adipokine levels and SHS subscores (that is, erosive and narrowing SHS). Here, we found that adiponectin level may predict joint erosive as well as narrowing disease progression at 1 year, which illustrates the long-term effect of this adipokine on all cell types within the RA joint [29-36].

We also found that the leptin level is a reliable surrogate biomarker of disease progression. The predictive value of leptin seems weaker than that of adiponectin, because we found no association of baseline leptin level and structural damage at inclusion of RA patients or subsequent rapid disease progression (∆SHS ≥5). Interestingly, two longitudinal studies obtained similar results: no predictive value of serum leptin level for radiographic disease progression [20,21].

Considering the direct link between serum adipokine levels and metabolic disorders such as obesity, type 2 diabetes, insulin resistance, or metabolic syndrome [37-39], our interpretation of serum adipokine levels by multivariate analysis included not only BMI, but also an estimate of insulin resistance by the HOMA-IR index was not considered in previous studies [18-21]. Likewise, we included the HOMA-IR index among the “metabolic confounders” in multivariate models. Interestingly, the association of adiponectin or leptin level and radiographic disease progression persisted in these models. Adiponectin level may be independent of fat-tissue status in exerting a deleterious effect on RA joints, and thus is not the sole biologic explanation for the protective effect of obesity on RA joint damage [40,41].

Treatment at baseline, especially corticosteroids, may modify adipokine levels [42,43]. In the ESPOIR cohort, most patients were untreated at the time of inclusion, but about 14% received steroids for a short time before inclusion. We included steroid prescription as a confounder in our multivariate analysis (model 4). Steroid use modulates adipokine levels themselves [42,43]. Interestingly, steroid prescription did not modify the association of serum adipokine levels and radiographic changes, so use of steroids, given to patients with more severe disease, cannot explain the link between adipokine levels and RA progression.

We found no association of visfatin/NAMPT levels and RA, which disagrees with the cross-sectional study of Rho *et al.*[19] but agrees with data from a recent longitudinal study [20]. Thus, the use of this adipokine as serum biomarker seems more elusive than adiponectin.

One limitation of our study is that some concerns may be raised by the definition of radiographic progression as SHS ≥1. However, to ensure the strength of our results, we performed two sensitivity analyses. First, we chose another more-stringent definition of radiographic progression (∆SHS ≥5), which corresponds to the destruction of one small rheumatoid joint, thereby delineating rapid radiographic progression [27]. With this definition, never used in previous studies on serum adipokines [20,21], the associations persisted, which suggests that adiponectin may accurately differentiate slow and fast disease progression. Second, we performed a linear regression analysis searching for a correlation between baseline adipokine levels as continuous variables and SHS values at 1 year. Baseline adiponectin again was associated with structural damage at 1 year.

Since we identified serum adiponectin level as a predictive marker of subsequent structural disease progression, we have attempted to determine the best threshold by using ROC curves analyses (4.14 μg/ml for ∆SHS ≥1 and 6.04 μg/ml for ∆SHS ≥5). These two cut-offs display good specificity (58% and 75% for ∆SHS ≥1 and ∆SHS ≥5, respectively) and high negative predictive value (75% and 92% for ∆SHS ≥1 or ∆SHS ≥5, respectively), especially for rapid radiographic progression. Thus, a serum adiponectin below these two thresholds may exclude the risk of structural progression.

The next step for the validation of serum adiponectin is to model a matrix that would combine serum total adiponectin with other markers (anti-CCP antibodies status, CRP level, baseline swollen-joint count, and erosions seen on radiography) [27]. Furthermore, even if serum adiponectin measurement has drawbacks, such as low sensitivity and positive predictive value, its predictive accuracy is in the same range as other recognized predictive markers of radiographic progression, according AUC (that is, DAS28 value, number of swollen joints, and CRP level). Finally, the independent association between serum adiponectin level and RA progression replicated here in such a large cohort gives pathophysiologic clues about the involvement of adipokines in the early clinical phase of RA and clearly demonstrates the link between a high level of serum adiponectin and structural disease progression.

## Conclusions

We demonstrated that serum adiponectin level at the time of RA diagnosis represents a surrogate biomarker of early radiographic disease progression in patients fulfilling the new ACR-EULAR 2010 criteria and thus may accurately identify patients at high risk of early joint destruction independent of inflammation, autoantibody status, and metabolic disturbances.

### Patient consent

Obtained.

### Ethics approval

Approval was obtained from Montpellier University Ethics Committee.

## Abbreviations

ACR: American College of Rheumatology; anti-CCP: Anti-cyclic citrullinated peptide; AUC: Area under the curve; BMI: Body mass index; CRP: C-reactive protein; ELISA: Enzyme-linked immunosorbent assay; ESR: Erythrocyte sedimentation rate; EULAR: European League Against Rheumatism; HAQ: Health Assessment Questionnaire; HOMA-IR: Homeostatic Model Assessment of Insulin Resistance; NAMPT: Nicotinamide phosphoribosyltransferase; OR: Odds ratio; RA: Rheumatoid arthritis; RF: Rheumatoid factor; ROC: Receiver-operated characteristic; SD: Standard deviation; SHS: Sharp/van der Heijde score; UA: Undifferentiated arthritis; ∆SHS: Change in total SHS.

## Competing interests

The authors declare that they have no competing interests.

## Authors’ contributions

Conception and design: MM, JS, JC, TS, and FB. Acquisition of data: SF, J-PB, and JC. Analyses and interpretation of data: MM, JS, SF, SK, J-PB, OM, FL, TS, JC, and FB. Drafting the manuscript: MM, JS, SK, and FB. Revising the manuscript: MM, JS, SF, SK, J-PB, OM, FL, TS, JC, and FB. All authors read and approved the final manuscript.

## Supplementary Material

Additional file 1: Figure S1 Association of baseline serum adipokine levels and radiographic joint erosive or narrowing disease progression defined as an increase of ≥1 in erosive or narrowing SHS between inclusion and 1 year. Click here for file

Additional file 2: Table S1 Association of serum adipokine levels and total Sharp/van der Heijde score (SHS) at 1 year. Click here for file
