# Supplementary material for: Serum level of adiponectin is a surrogate independent biomarker of radiographic disease progression in early rheumatoid arthritis: results from the ESPOIR cohort
Source: Arthritis Res Ther. 2013 Dec 9;15(6):R210. doi: 10.1186/ar4404 (PMC3978925; doi:10.1186/ar4404)
Supplement: Additional file 1: Figure S1 — Association of baseline serum adipokine levels and radiographic joint erosive or narrowing disease progression defined as an increase of ≥1 in erosive or narrowing SHS between inclusion and 1 year. [file ar4404-S1.ppt]

## Slide 1
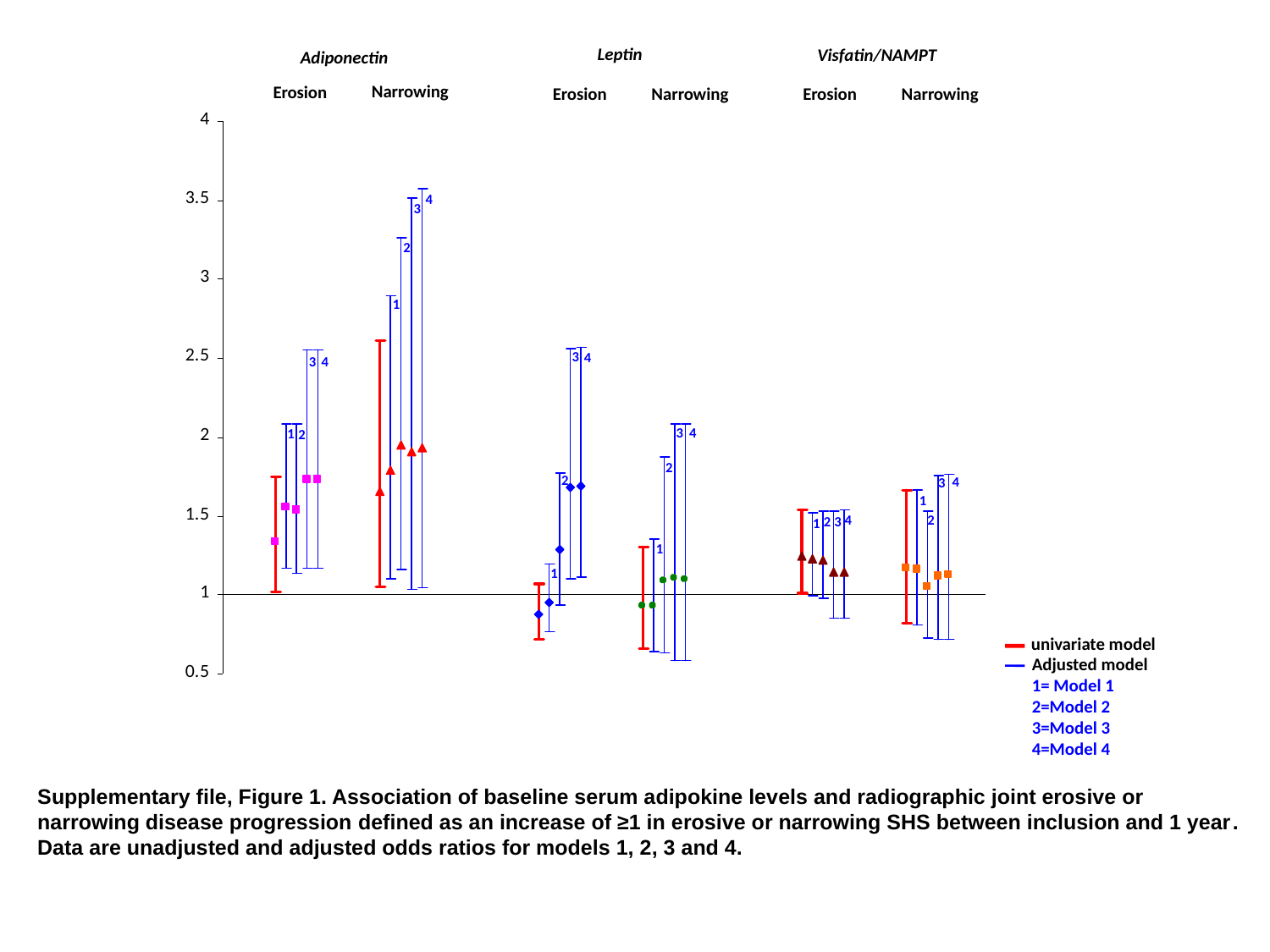

Leptin
Visfatin/NAMPT
Adiponectin
Narrowing
Erosion
Erosion
Narrowing
Erosion
Narrowing
4
3
2
1
3
4
3
4
3
4
1
2
2
2
4
3
1
2
4
3
2
1
1
1
univariate model
Adjusted model
1= Model 1
2=Model 2
3=Model 3
4=Model 4
Supplementary file, Figure 1. Association of baseline serum adipokine levels and radiographic joint erosive or narrowing disease progression defined as an increase of ≥1 in erosive or narrowing SHS between inclusion and 1 year.
Data are unadjusted and adjusted odds ratios for models 1, 2, 3 and 4.
