# Supplementary material for: Serum level of adiponectin is a surrogate independent biomarker of radiographic disease progression in early rheumatoid arthritis: results from the ESPOIR cohort
Source: Arthritis Res Ther. 2013 Dec 9;15(6):R210. doi: 10.1186/ar4404 (PMC3978925; doi:10.1186/ar4404)
Supplement: Additional file 2: Table S1 — Association of serum adipokine levels and total Sharp/van der Heijde score (SHS) at 1 year. [file ar4404-S2.doc]

**Supplementary files.**

**Supplementary file, Table 1. Association of serum adipokine levels and total Sharp/van der Heijde score (SHS) at 1 year.**

Multivariate models:

Model 1 (“base”): sex + age

Model 2 (“metabolic”): Model 1 + BMI + HOMA-IR index

Model 3 (“metabolic + RA”): Model 2 + CRP level + DAS28-ESR value + HAQ score + RF status + anti-CCP antibody status + presence of radiographic changes at inclusion

Model 4 (“metabolic + RA + steroid”): Model 3 + steroid prescription at inclusion

The following data were log-transformed: CRP level, HAQ score and serum adipokine levels

|  | **Univariate model** | | **Model 1 “Base”** | | **Model 2 “metabolic”** | | **Model 3 “metabolic + RA“** | | **Model 4 “metabolic + RA + steroid“** | |
| --- | --- | --- | --- | --- | --- | --- | --- | --- | --- | --- |
|  | **β** | **p** | **β** | **p** | **β** | **p** | **β** | **p** | **β** | **p** |
| **Adiponectin** | **0.08** | **0.02** | **0.11** | **0.0006** | **0.11** | **0.0009** | **0.13** | **0.003** | **0.13** | **0.0026** |
| **Leptin** | -0.03 | 0.54 | -0.01 | 0.80 | -0.004 | 0.90 | 0.01 | 0.74 | 0.01 | 0.73 |
| **Visfatin/NAMPT** | 0.02 | 0.68 | 0.02 | 0.66 | 0.001 | 0.98 | 0.008 | 0.89 | 0.008 | 0.89 |
